# Facile and cost-effective technique to control europium oxidation states in glassy fluorophosphate matrices with tunable photoluminescence

**DOI:** 10.1038/s41598-022-21981-z

**Published:** 2022-11-05

**Authors:** Agata Jarocka, Bartosz Fetliński, Paweł Dębowski, Tomasz K. Pietrzak, Kacper Jurak, Marek Wasiucionek

**Affiliations:** 1 Faculty of Physics, Warsaw University of Technology, Koszykowa 75, 00-662 Warsaw, Poland; 2 Faculty of Electronics and Information Technology, Institute of Microelectronics and Optoelectronics, Warsaw University of Technology, Koszykowa 75, 00-662 Warsaw, Poland; 3 Department of Electrochemistry, Corrosion and Material Engineering, Gdansk University of Technology, Narutowicza 11/12, 80-233 Gdansk, Poland

**Keywords:** Optical physics, Optical materials and structures, Applied optics, Materials for optics

## Abstract

Inorganic fluorophosphate glasses doped with Eu$^{2+}$/Eu$^{3+}$ are potential candidates for phosphors for commercial white LEDs. This report presents a fast, inexpensive and effective method of controlling the relative concentrations of Eu$^{2+}$/Eu$^{3+}$ photoluminescent centers in these glasses. The technique consists of a fast quenching of the melt of initial reagents under appropriate conditions. Eu$^{2+}$/Eu$^{3+}$ ratio was controlled by carrying out the melting under a reducing atmosphere at a temperature between 1000 and 1200 $^\circ$C for periods of 5 to 15 minutes. The reducing atmosphere was provided by a ’double crucible’ technique and did not require special gas lines during the synthesis. The samples were studied by several complementary experimental methods (X-ray diffractometry—XRD, X-ray photoelectron spectroscopy—XPS, photoluminescence—PL—and photoluminescence excitation—PLE—spectroscopies as well as optical transmission spectroscopy). It was shown that the syntheses resulted in amorphous materials with different relative Eu$^{2+}$/Eu$^{3+}$ concentration ratios, strongly dependent on the preparation conditions: the temperature and the time of melting in a reducing atmosphere. Moreover, changes in these ratios strongly affected the materials’ PL and PLE spectra. Demonstration of reproducible smooth transition from amaranth to blue luminescence color, with white in between, was the most spectacular result of this work.

## Introduction

Photoluminescence (PL) of inorganic matrices doped with rare-earth (RE) elements was discovered more than 150 years ago^1,2^. Since then, the knowledge and understanding of the PL phenomena in those materials have progressed immensely. As a result, it seems that the basic mechanisms of PL in that group of materials are satisfactorily explained (e.g.^3,4^). In particular, it is well established that the photoluminescence spectra of RE ions inside given matrices depend not only on the characteristic electronic structures of RE ions themselves, but also on the matrices and the local surrounding of RE ions in these matrices^3,4^.

The scientific interest in PL materials doped with RE ions remains high due to their applications, mainly as phosphors in artificial lighting, optical displays, screens, etc.^4–6^. Their use in white LEDs (wLED) is significant and essential for indoor and outdoor solid-state lighting (SSL) development. The main advantage of today’s commercial wLEDs is their high luminous efficacy (often exceeding 100 lm/W, compared to ca. 13 lm/W for traditional incandescent bulbs^7^), which makes them very energy-efficient light sources with long lifespans^8,9^. These factors (high luminous efficacy, long lifespan, and decreasing fabrication costs) are significant from the economic and ecological viewpoints. Artificial lighting consumes almost 20% of global electric energy^10,11^ and considerably contributes to CO$_2$ emissions. It is predicted that until 2030, the ongoing massive replacement of traditional light sources by wLEDs can decrease the global energy consumption in the lighting industry by more than 30% compared to 2017^12^. One of the major challenges related to wLEDs is to liken their emission spectra to that of natural light, at possibly low cost, to make them affordable and safe^13,14^. That is important because prolonged exposure of human eyes to the ’unnatural’ white light of most of today’s commercial wLEDs may cause eyes fatigue and can even lead to irreversible vision dysfunction, poor sleeping, and other negative consequences for health^13,15,16^.

Many materials and synthetic routes have been proposed leading to phosphors which could convert blue/near UV excitation light of InGaN chips into a white light resembling the daylight^11,17^. Europium is one of the most often used RE elements exhibiting photoluminescence tested in phosphors for wLEDs^18^. It can be present in two oxidation states: Eu$^{3+}$ and Eu$^{2+}$. Both ions exhibit strong luminescence in the visible range, but in different parts thereof: Eu$^{3+}$ mostly in the orange-red range, and Eu$^{2+}$ usually in blue-green, but easily extendable, by a proper choice of a matrix, to cover the whole visible range. Therefore, by the appropriate mixing of photoluminescence spectra of both types of Eu centers, one can ‘tailor’ the effective emission of a Eu$^{2+}$/Eu$^{3+}$ couple in an appropriate matrix and make the resulting PL light similar to daylight. In several earlier papers on phosphors with mixed Eu$^{2+}$/Eu$^{3+}$ PL centers, the researchers have used either crystalline matrices, such as NaAlSiO$_4$, Na$_5$Gd$_9$F$_{32}$^19^, Ca$_4$Si$_2$O$_7$F$_2$^20^ or amorphous ones like strontium borate glasses^21^ or Al$_2$O$_3$-SiO$_2$ glasses^22^. In the literature, one can also find attempts to control the photoluminescence spectrum by mixing dopants, for example, Eu$^{3+}$ with Tb$^{3+}$^23^.

Usually, the control of Eu$^{2+}$/Eu$^{3+}$ proportions in phosphors based on crystalline matrices requires relatively long and complicated technological processing^20^. We propose to apply a much simpler and cost-effective synthesis route. The idea involves preparation of glasses based on matrices of the NaF-Al$_2$O$_3$-P$_2$O$_5$ system, doped with Eu$_2$O$_3$. The main part of the processing consists of melting the initial mixtures under specific conditions: in the reducing atmosphere, at high temperature (between 1000 and 1200 $^\circ$C), and for relatively short periods (up to 15 min). This stage is followed by the fast quenching of the melts. The exposure of the melts to the reducing atmosphere inside the oven leads to a gradual controllable reduction of europium from its initially predominant Eu$^{3+}$ fraction to a Eu$^{2+}$/Eu$^{3+}$ mixture. The glassy matrix of the NaF-Al$_2$O$_3$-P$_2$O$_5$ system was chosen because such matrices are inexpensive, relatively easy to prepare, have a low refraction index and, in general, spectra of given RE ions in amorphous matrices are broader than crystalline ones^24,25^. Moreover, the selected chemical composition of the glass corresponds to that of a Na$_3$Al$_2$(PO$_4$)$_2$F$_3$ compound whose crystalline structure is known^26^. It is also known that various crystalline phosphates and fluorophosphates have been already successfully used as phosphors for white LEDs^14,27^. The proposed method is a simple process in which the setup does not require any gas line to ensure a reducing atmosphere. Instead, a simple ‘double-crucible’ technique is used^28^. This technique had been successfully used by our group to control oxidation states of vanadium and iron in glassy-crystalline nanomaterials—potential cathode candidates for Li-ion and Na-ion batteries^29,30^. It was also used in our preliminary work on glassy Eu$^{2+}$/Eu$^{3+}$ phosphors^31^.

## Results

### Synthesis

In this study, we synthesized ten glassy batches, all based on NaF–Al$_2$O$_3$–P$_2$O$_5$ glasses doped with 1 wt% of Eu$_2$O$_3$ but prepared at different conditions. One was prepared in air, all others in a reducing atmosphere. The list of the synthesized samples is given in Table 1.

**Table 1:** List of the synthesized samples with the synthesis parameters (atmosphere, melting temperature and time).

Sample	Atmosphere	Temperature [$^\circ$C]	Time of melting [min]
N-1200-15	Air	1200	15
R-1000-5	Reducing	1000	5
R-1000-10	Reducing	1000	10
R-1000-15	Reducing	1000	15
R-1100-5	Reducing	1100	5
R-1100-10	Reducing	1100	10
R-1100-15	Reducing	1100	15
R-1200-5	Reducing	1200	5
R-1200-10	Reducing	1200	10
R-1200-15	Reducing	1200	15

The synthesis details are described in the “Methods” section.

### X-ray diffractometry (XRD)

**Figure 1:**
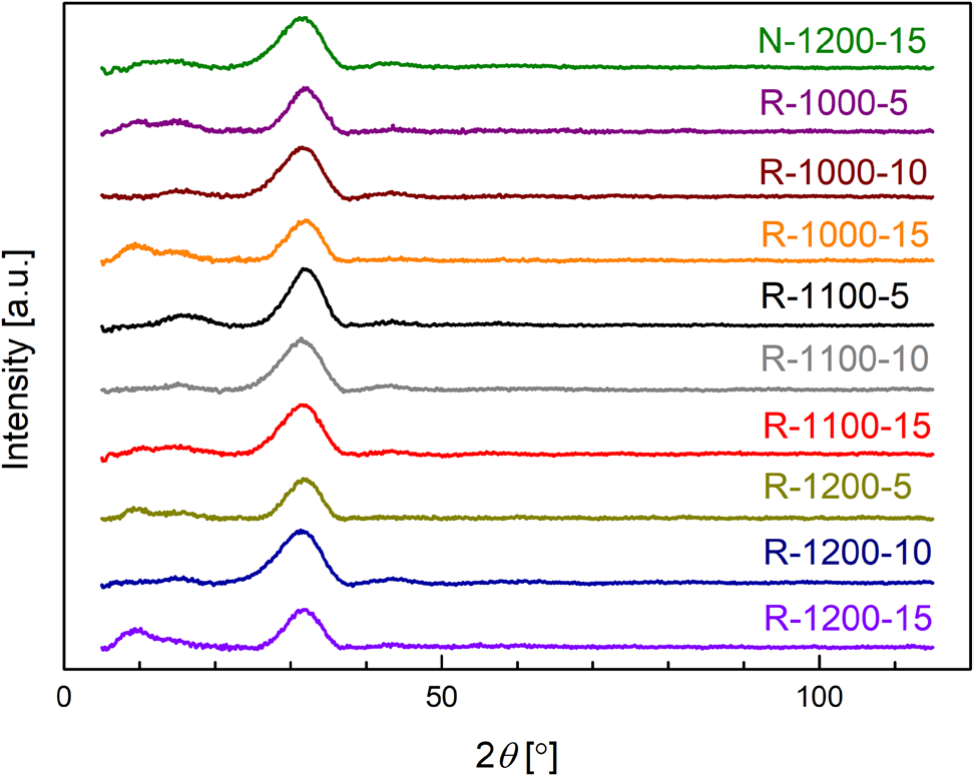
XRD patterns of all synthesized materials. The labels denote the preparation conditions: the atmosphere (N-air, R-reducing atmosphere), temperature of the melting stage (in $^\circ$C) and its duration (in min, cf. Table 1).

The starting point of this work was to synthesize and study glassy phosphors containing various proportions of Eu$^{2+}$/Eu$^{3+}$ PL centers. The amorphous state of as-prepared materials was examined by X-ray diffractometry (XRD). The XRD patterns of all synthesized compositions are shown in Fig 1. All of them contain a wide halo centered at ca. 31$^\circ$ and do not show any presence of Bragg reflections. This proves that all synthesized materials, regardless of their preparation conditions, were fully amorphous.

### Photoluminescence spectroscopy (PL)

**Figure 2:**
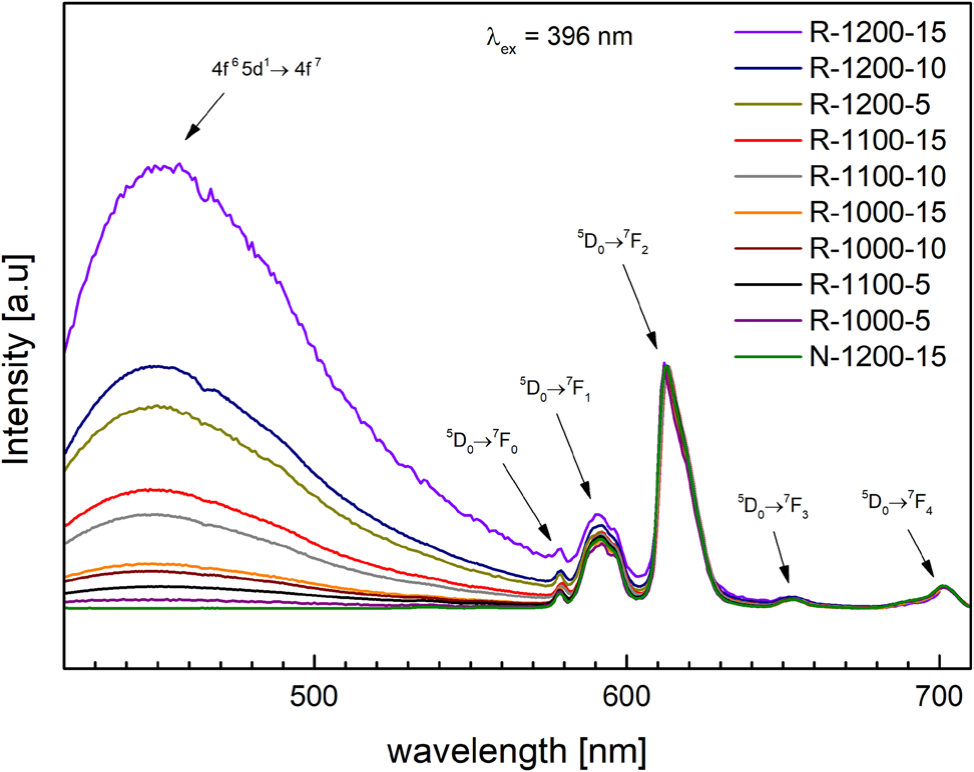
Photoluminescence spectra with transitions described in the figure. Samples were excited with 396 nm wavelength.

One of the main goals of this study was to monitor the effect of varying relative contents of Eu$^{2+}$ and Eu$^{3+}$ in the glassy matrices on their photoluminescence spectra. In this work, the excitation wavelength was set to $\lambda _{ex}=396$ nm because the light of that (and similar) wavelength activates the photoluminescence of both Eu$^{2+}$ and Eu$^{3+}$ centers^8^. Figure 2 presents the PL spectra of all samples under study. All characteristic features of the PL spectra were identified and ascribed to well-known electronic-vibrational transitions of either Eu$^{2+}$ or Eu$^{3+}$ ions.

Emission spectra are characterized by relatively broad and unresolved peaks, especially in comparison with ones observed in crystalline structures. Since such broad lines immediately raise the question of correct measurement techniques, special attention was given to this phenomenon to ensure that the spectra are measured in an optimal experimental setup, balancing signal level and spectral resolution. Measurements with narrow slits were conducted to check if it is possible to resolve the Eu peaks further. Yet, even with highly narrow slits, resolving the peaks was impossible. Thus, the broad emission is not a measurement artifact but a physical phenomenon caused by the glassy character of the materials.

The broad peak centered at approx. 460 nm, whose intensity strongly depends on the synthesis conditions, corresponds to the optical transitions of Eu$^{2+}$: $4f^65d^1\rightarrow 4f^7$^20,21,32^. These are allowed transitions in which the energy of 5*d* state of Eu$^{2+}$ is very sensitive to the local crystal field and is split into $t_{2g}$ and $e_g$ orbitals^33^. Therefore, the emission of Eu$^{2+}$ is composed of many overlapping transitions which lead to a broad emission band visible in Fig. 2.

On the other hand, sharp PL peaks above 550 nm are related to the transitions between $4f - 4f$ electronic–vibrational states of Eu$^{3+}$ ions: $^5D_0 \rightarrow ^7F_0$ at 580 nm, $^5D_0 \rightarrow ^7F_1$ at 592 nm, $^5D_0 \rightarrow ^7F_2$ at 612 nm, $^5D_0 \rightarrow ^7F_3$ at 654 nm and $^5D_0 \rightarrow ^7F_4$ at 701 nm^16,21,22,34,35^. These peaks weakly depend on the synthesis, and therefore, their fixed positions, observed in the present study, as well as many others, are consistent with the fact that 4*f* shell of europium is strongly shielded from the surrounding by its outer 5*s* and 5*p* electrons^36^. Based on the relative intensity of the emission lines of europium in different oxidation states, it can be deduced that along with the increase of time and temperature of the synthesis in an oxygen-free atmosphere, Eu$^{3+}$ ions are gradually reduced to Eu$^{2+}$. In the case of the sample N-1200-15, which was synthesized at high temperature (1200 $^\circ$C) for a long time (15 min) in air, there is no visible trace of a broad $4f^65d^1\rightarrow 4f^7$ transition centered at ca. 460 nm, characteristic for Eu$^{2+}$ photoluminescence. This band starts to be visible and increases in intensity with increasing temperature and time when the melting was carried out in a reducing atmosphere. Its height and area are the highest for the material R-1200-15. These observations confirm once more that this band is entirely due to photoluminescence of Eu$^{2+}$ centers, whose relative fraction increases during a prolonged synthesis in a reducing atmosphere.

Taking into account that the PL band of Eu$^{2+}$ is strongly affected by the syntheses conditions and PL lines of Eu$^{3+}$ are relatively insensitive to these conditions, it can be concluded that by setting appropriate synthesis parameters (atmosphere, temperature and time), one can control the effective PL spectra of mixed Eu$^{2+}$/Eu$^{3+}$ centers and to liken them to those of daylight.

**Figure 3:**
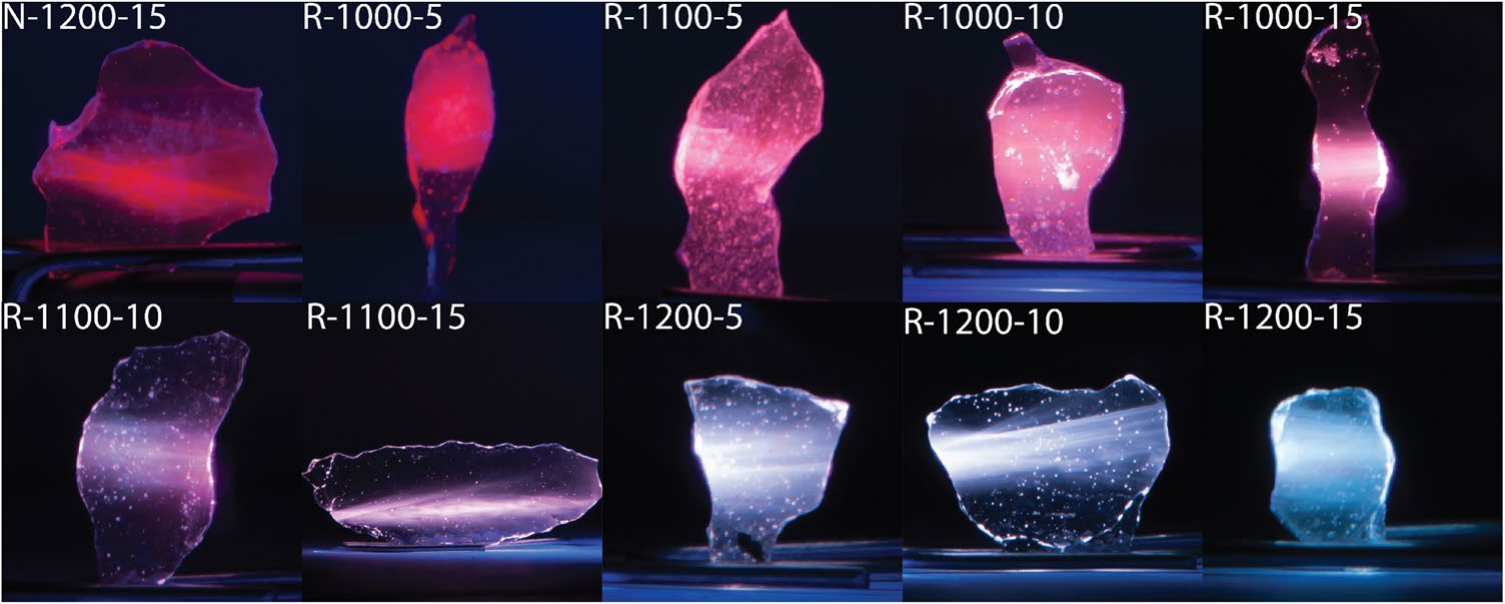
Photographs of the obtained samples illuminated with 405 nm laser.

**Figure 4:**
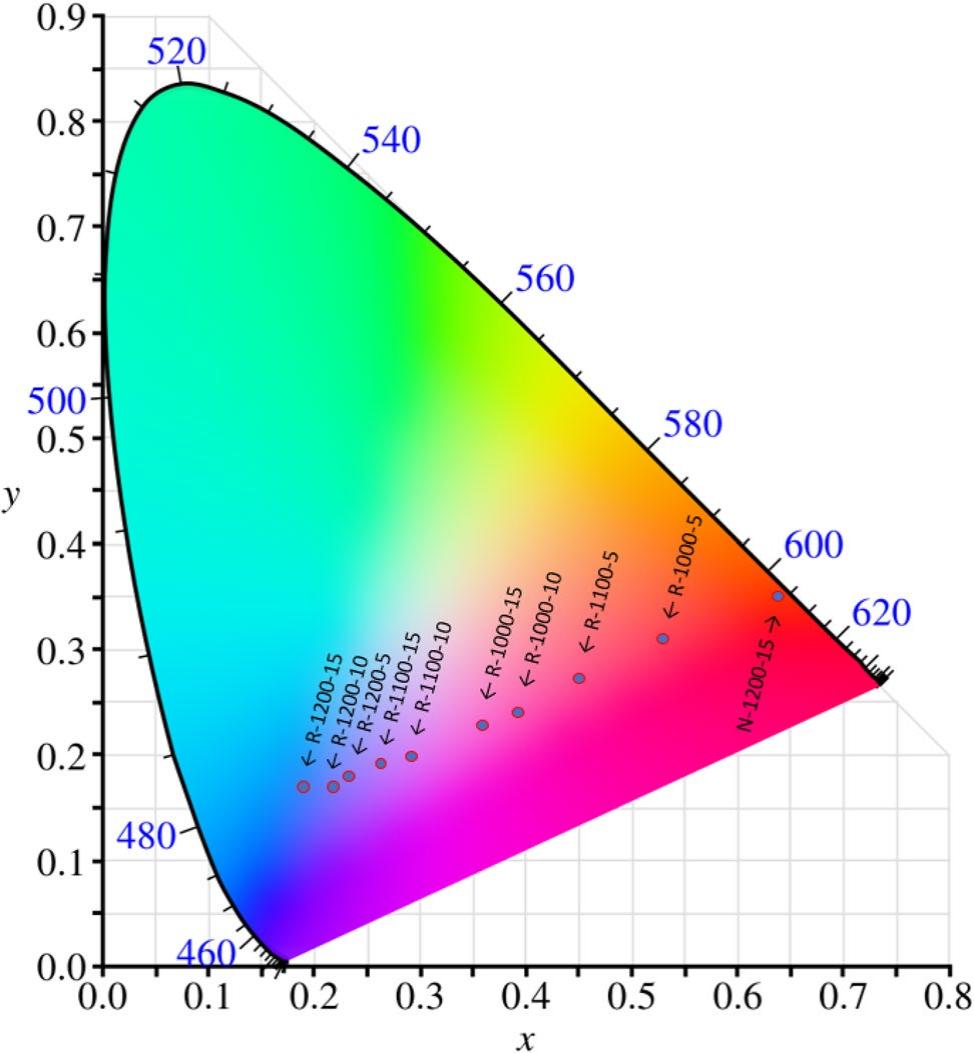
CIE 1931 color space chromaticity diagram with marked samples’ coordinates. Diagram adapted from^37^.

A set of photographs presented in Fig. 3 shows how the conditions of syntheses affect the visual impression of PL spectra of as-prepared glasses. The excitation light wavelength used in all cases in Fig. 3 was set to 405 nm. For a sample prepared in the air (N-1200-15), the effective color of photoluminescence is red. Then for samples held in a reducing atmosphere at progressively higher temperature and longer periods, the color changes first to violet/purple (R-1100-10), to whitish (R-1200-10), and finally to white-bluish (R-1200-15). A gradual change of colors is also visible when converting spectra to CIE color space coordinates (Fig. 4). Calculated coordinates decrease with increased time and temperature of the synthesis—values shown in Table 2. However, it is worth mentioning that photoluminescence spectra are collected from 420 nm, and standard observer functions start in lower wavelengths. Therefore, values (especially those in more blueish regions) could shift slightly left and up (lower x and higher y value) if lower wavelength were considered.

All shown changes in photoluminescence emission and CIE color space coordinates are due to the increasing share of Eu$^{2+}$ ions at the expense of Eu$^{3+}$ caused by different synthesis conditions.

**Table 2:** List of the calculated CIE color space coordinates.

Sample	x	y
N-1200-15	0.64	0.35
R-1000-5	0.53	0.31
R-1100-5	0.45	0.27
R-1000-10	0.39	0.24
R-1000-15	0.36	0.23
R-1100-10	0.29	0.20
R-1100-15	0.26	0.19
R-1200-5	0.23	0.18
R-1200-10	0.22	0.17
R-1200-15	0.19	0.17

### Photoluminescence excitation spectroscopy (PLE)

**Figure 5:**
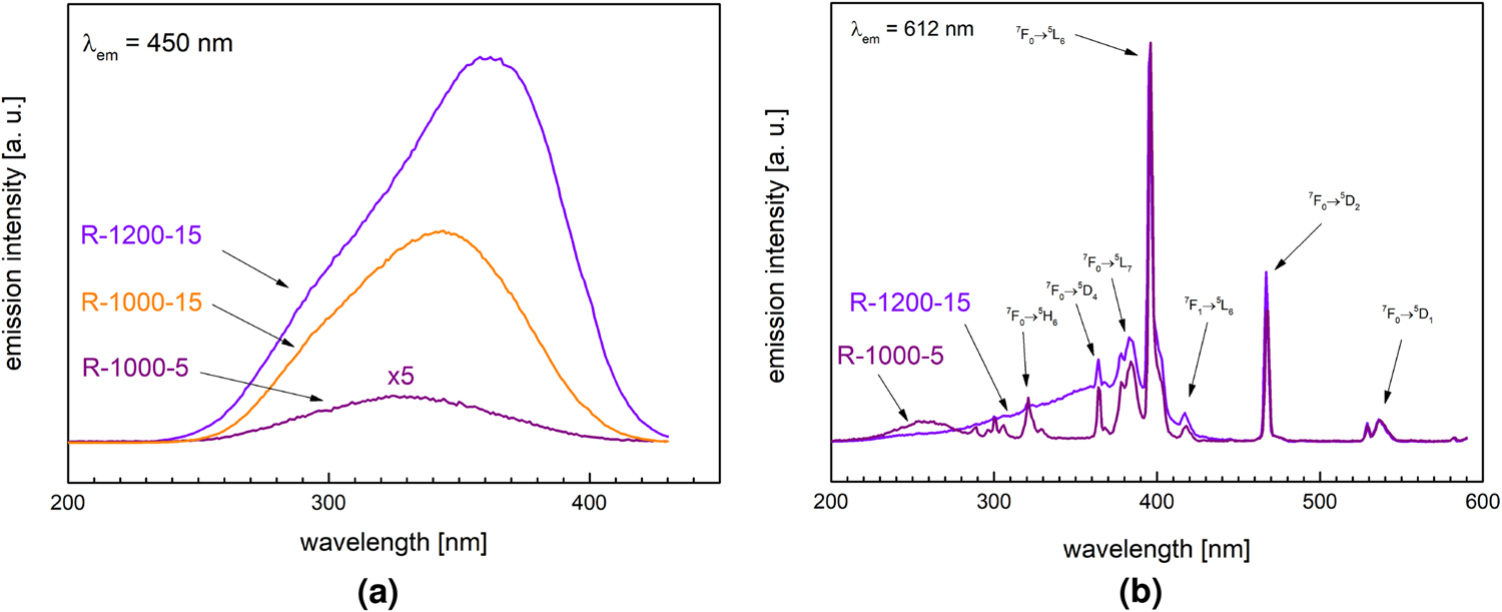
PLE spectra of selected samples: (**a**) for $\lambda _{em}=450$ nm, (**b**) $\lambda _{em}=612$ nm.

Photoluminescence excitation spectroscopy measurements have been carried out to complement the information obtained from PL spectra on optical transitions of europium centers. The results of PLE measurements are shown in Fig. 5a,b.

In Fig. 5a the emission wavelength was set to $\lambda _{em} = 450$ nm, in the proximity of the maximum of PL spectra of Eu$^{2+}$ centers (cf. Fig. 2). The figure shows the excitation spectra of three samples (R-1000-5, R-1000-15 and R-1200-15) synthesized in a reducing atmosphere at low temperature (R-1000-5 and R-1000-15) and high temperature (R-1200-15). In all cases, the excitation spectra form a wide smooth band whose intensity increases with temperature and time. Such spectra are entirely due to Eu$^{2+}$ centers, whose concentration increases with the temperature and time of the melting in a reducing atmosphere. An apparent shift of the band maximum towards longer wavelengths should be attributed to the increasing fraction of Eu$^{2+}$ and its contribution to the PLE spectra.

The PLE spectra for the $\lambda _{em} = 612$ nm, shown in Fig. 5b for samples R-1000-5 and R-1200-15, correspond to Eu$^{3+}$ and are much more complicated. Nearly all sharp features were identified and labeled in the figure. They correspond to Eu$^{3+}$ transitions between their initial states of $^7F_0$ or $^7F_1$ to a series of $^5D_J$ ($J=0,1,2,3,4$) and $^5L_7$, $^5L_6$ (the strongest one) and $^5H_6$. The positions of these lines agree with those reported in the literature for PLE of Eu$^{3+}$ in various matrices (e.g. LiGd$_5$P$_2$O$_{13}$^38^).

### Optical transmission

**Figure 6:**
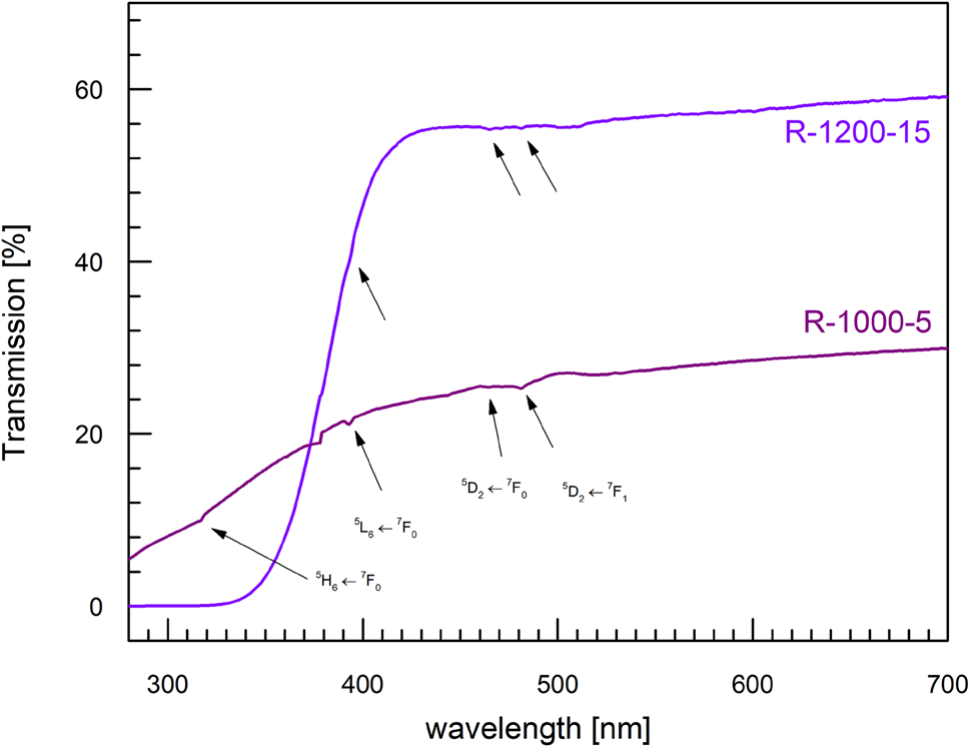
Transmission spectra of two samples: R-1200-15 (purple line) and R-1000-5 (violet line). Characteristic electron transitions of Eu$^{3+}$ ions were identified, marked with arrows and annotated.

The optical transmission spectra of two samples (R-1000-5 and R-1200-15) in the range 280–700 nm are shown in the Fig. 6. Both samples are transparent in the visible range, but one can notice significant differences between their transmission spectra. First of these differences is the absolute value of the transmission coefficient at longer wavelengths: for the R-1200-15 material, it is around 55–60 % and for the R-5-1000 one, it is much lower, ca. 25–30 %. The second difference is observed at shorter wavelengths in the UV range, where the sample R-1000-5 is still transparent, and the R-1200-15 one becomes opaque for wavelengths below ca. 340 nm. We have attributed the latter difference to the Eu reduction process being much more advanced in the R-1200-15 material (higher temperature, longer melting in a reducing atmosphere). The higher content of Eu$^{2+}$ in the material means stronger absorption in the near UV range, which is characteristic for Eu$^{2+}$ centers^39^. Moreover, on quite smooth transmission spectra one can see several small ‘kinks’ which have been attributed to the electronic transitions of Eu$^{3+}$ : $^5L_6 \leftarrow ^7F_0$ (393 nm), $^5D_2 \leftarrow ^7F_0$ (465 nm) and $^5D_2 \leftarrow ^7F_1$ (481 nm). Very similar positions of these transitions (393 nm, 464.5 nm) were reported by Binnemans et al.^40^ for phosphors based on 75NaPO$_3$-20CaF$_2$-5EuF$_3$. These features are visible for both samples but are slightly stronger for the sample prepared at a lower temperature and shorter time (R-1000-5). This observation is consistent with the higher content of Eu$^{3+}$ in that sample. The above-mentioned transitions are in perfect agreement with transitions seen in PLE spectra shown in Fig. 5b. The slight shift in the spectrum at ca. 380 nm is a reproducible experimental artifact due to the change of optical settings of the apparatus.

### X-ray photoelectron spectroscopy (XPS)

XPS experiments were carried out on all samples under study to determine relative concentrations of Eu$^{2+}$/Eu$^{3+}$ in the samples prepared under different conditions. However, due to the low concentration of europium in the samples’ composition, some spectra exhibited a poor Eu signal-to-noise ratio, making it difficult to perform quantitative analysis. Taking into consideration that the relative ratio of Eu$^{2+}$/Eu$^{3+}$ does not have to change significantly to cause large changes in optical properties and aforementioned signal intensity, it was decided to focus on 5 samples (Table 3). All samples fit one model for both carbon and europium^41^. The calibration of the energy scale was based on the neutral carbon C 1s (284.8 eV). Accordingly, the peaks of Eu$^{3+}$ and Eu$^{2+}$ are located at 1135 eV and 1125 eV, respectively. The fit was made based on the literature^41^.

**Figure 7:**
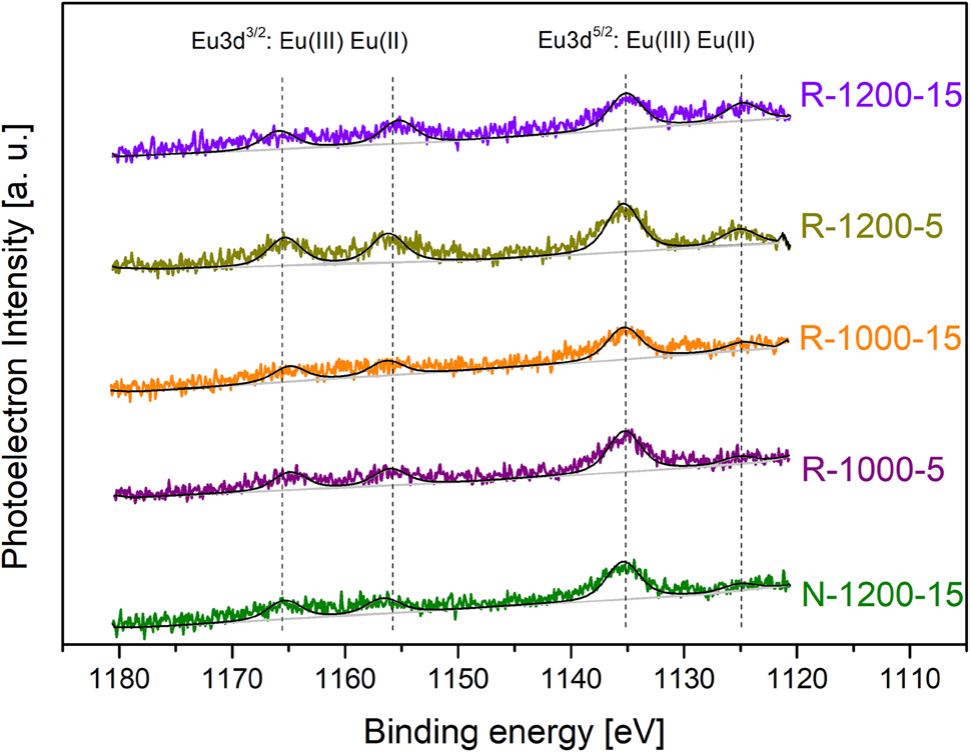
The Eu3d high-resolution XPS spectra of investigated samples. Signals from samples are marked with respected colors and the fits are represented by gray or black lines.

The collected XPS spectra are shown in Fig. 7 and calculated Eu$^{2+}$/Eu$^{3+}$ ratios are presented in Table 3. Due to the low XPS signal-to-noise ratio of Eu, the values presented in the table should be considered qualitatively rather than strictly quantitatively. Nevertheless, these results confirm that by applying reducing atmosphere, sufficiently high temperature and appropriate melting time, it is possible to cause a controlled partial reduction of Eu$^{3+}$ to Eu$^{2+}$. This gradual reduction of Eu$^{3+}$ to Eu$^{2+}$ is responsible for changes in the optical properties of the samples described in previous sections. However, it does not change the vitreous character of these materials. Additionally, when comparing these results with photoluminescence spectra (Fig. 2) and photographs of blue laser-excited samples (Fig. 3), one can notice huge differences in the PL spectra and the visual appearance of the emitted light. It should be emphasized that even a moderate change of relative Eu$^{2+}$/Eu$^{3+}$ ratios can result in a considerable change in PL spectra. As presented in the Table 3, the ratio changes from 0.17 for the least reduced sample to 0.35 for the most reduced one. This corresponds to an increase in the relative content of Eu$^{2+}$ from 14.6 (sample N-1200-15) to 26.1% (sample R-1200-15). The relative Eu$^{3+}$ content decreases accordingly.

**Table 3:** Eu$^{2+}$ to Eu$^{3+}$ ratio calculated from XPS measurements.

Sample name	Eu$^{2+}$ [%]	Eu$^{3+}$ [%]	Eu$^{2+}$/Eu$^{3+}$
R-1200-15	26.1	73.9	0.35
R-1200-5	23.8	76.2	0.31
R-1000-15	19.8	80.2	0.25
R-1000-5	14.8	85.2	0.17
N-1200-15	14.6	85.4	0.17

## Discussion and conclusions

In this work, 10 glassy NaF–Al$_2$O$_3$–P$_2$O$_5$ matrices doped with 1 wt% Eu$_2$O$_3$ were successfully synthesized with the melt-quenching method. Nine samples were melted in a reducing atmosphere at different temperatures (1000 $^\circ$C, 1100 $^\circ$C and 1200 $^\circ$C) and different times (5, 10 and 15 min). One sample was synthesized in the air at 1200 $^\circ$C for 15 min. This way, a wide range of various atmosphere/temperature/time configurations was obtained.

To determine vitreous character of samples, X-ray diffractometry was used. All diffractograms were characterized by a wide amorphous halo at ca. 31$^\circ$. Moreover, no visible Bragg reflections were observed. Therefore, all samples were amorphous, regardless of synthesis conditions.

Photoluminescence spectroscopy results show changes between samples’ emissions spectra when excited with 396 nm (Fig. 2). All observed electronic transitions were ascribed to either Eu$^{3+}$ (^16,21,22,34,35^) or Eu$^{2+}$ ions (^20,21,32^) characteristic ones. Eu$^{3+}$ ions are characterized by a sharp peak above 550 nm, while Eu$^{2+}$ by a broad band centered at ca. 460 nm. Analyzing the changes in peaks’ intensities—a gradual increase of the broad band at 460 nm in comparison to the sharp peak in 612 nm, one can conclude that appearance of Eu$^{2+}$ ions is higher for samples synthesized in a more reducing atmosphere. What is more, both time and temperature have a strong impact on this phenomenon. These spectra, without a doubt, prove that applying reducing atmosphere and increasing time and temperature of synthesis cause a reduction of europium in the 3+ oxidation state to the one in 2+. It is also visible ’to the naked eye’ in Fig. 3 where samples were illuminated with a blue laser. Their photoluminescence color gradually changes with synthesis conditions from red, through whitish, to light blue. The observed color changes can be quantitatively represented in the CIE chromacity coordinates (Fig. 4). It can be seen that the experimental points lie along a straight line in that diagram. This strongly suggests that by optimizing the temperature and duration of the syntheses one can effectively liken the color of PL emissions to that of the daylight.

Photoluminescence excitation spectroscopy and optical transmission measurements were carried out as complementary methods to confirm the dependence of optical properties of the studied materials on a gradual change in Eu$^{2+}$/Eu$^{3+}$ ratio. The results are in a good agreement with those of PL spectroscopy. Moreover, they also show that the glasses obtained in reducing atmosphere with higher temperature and melting time are characterized by higher absorption in the UV region. The fact that the absorption coefficients in UV range are higher for Eu$^{2+}$ than for Eu$^{3+}$ has been reported for several europium doped systems^38^. This additionally supports our conclusions about the effect of Eu reduction on the optical properties of the studied glasses.

XPS studies were in agreement with the aforementioned results. The relative concentration of Eu$^{2+}$/Eu$^{3+}$ ions in the selected samples was determined (Fig. 7) and, despite high uncertainties due to low signal-to-noise ratio, indicates a significant increase in this ratio along with the time and temperature of materials synthesis in the reducing atmosphere. These results were combined and presented in Table 3. In particular, the Eu$^{2+}$/Eu$^{3+}$ ratio for the sample (R-1200-15) was approximately twice as high as that for the sample prepared in air (N-1200-15).

Presented studies prove that the proposed facile route of the synthesis leads to glassy phosphors with the tunable color of photoluminescence. This is due to the fact that the reducing atmosphere, higher temperature, and longer synthesis time cause reduction of europium ions from Eu$^{3+}$ to Eu$^{2+}$. All presented results indicate that by following that route one can produce glassy phosphors for white LEDs which spectra can be tuned to imitate the daylight. In the future, the use of such phosphors in commercially produced LEDs can improve the quality of lightning that would not harm human vision. On the contrary, they would be healthy for our eyes. In terms of future use in industry, it should be appreciated that the proposed method of controlling oxidation states is simple, inexpensive, scalable and based on well-known processes.

## Methods

Batches of 10 glassy matrices of the system NaF–Al$_2$O$_3$–P$_2$O$_5$, of the nominal composition corresponding to Na$_3$Al$_2$(PO$_4$)$_2$F$_3$, doped with 1 wt% Eu$_2$O$_3$ were prepared using a standard melt-quenching method. Appropriate proportions of pre-dried starting reagents: Al$_2$O$_3$ (POCh, p.a.), NH$_4$H$_2$PO$_4$ (POCh, 99,5%) were mixed together and ground with a mortar and a pestle. Then the mixtures were calcined in air at 240$^\circ$C in a programmable furnace to remove volatile products of the thermal decomposition of the starting compounds. The next stage consisted in the admixture of the appropriate amounts of NaF (Sigma-Aldrich, >99%) and 1 wt % of Eu$_2$O$_3$ (POCh, 99.9%), to the previously synthesized and ground material. Then the mixtures were ground again, put into ceramic crucibles and melted in an induction furnace Argenta AFI-02 at temperatures 1000 $^\circ$C, 1100 $^\circ$C or 1200$^\circ$C (see Table 1). The duration of melting was set to 5, 10 or 15 min (Table 1). One batch out of 10 was melted in an oxidizing atmosphere (air), whereas 9 others were melted under a reducing atmosphere, using a double-crucible arrangement^29,31^. The reducing atmosphere was provided by filling outer crucible with charcoal which while burning at low oxygen concentration creates carbon monoxide. That provides reducing conditions for europium ions. Additionally, the crucibles were closed to enhance the reduction process and prevent fluorophosphate from evaporation. Finally, the melts were quickly poured onto a stainless-steel plate held at room temperature and immediately pressed from above by another identical plate to ensure fast enough cooling. Below, the chemical equation associated with reduction process is provided:

1 $$\begin{aligned} \hbox {Eu}_{2}\hbox {O}_{3} + \hbox {CO} = 2 \,\hbox {EuO} + \hbox {CO}_{2} \end{aligned}$$

As-received samples were characterized with the following complementary experimental methods: X-ray diffractometry (XRD), photoluminescence spectroscopy (PL), photoluminescence excitation spectroscopy (PLE), transmission optical spectroscopy and high-resolution X-ray photoelectron spectroscopy (XPS). All measurements were performed at room temperature.

The powder X-ray diffractometry method was used mainly to confirm the amorphous character of the as-synthesized samples. Samples were powdered before measurements. These studies were carried out using a PANalytical Empyrean X-ray diffractometer (with copper lamp of the wavelength Cu$_{K\alpha }$ = 1.54 Å with working parameters: $U=40$ kV, $I=35$ mA).

The optical transmission spectra of the samples were acquired using a dual channel Perkin Elmer Lambda 950 spectrometer. The emission (PL) and excitation (PLE) spectra were collected using a Photon International spectrometry (PTI) setup with a xenon lamp source, double Bragg gratings monochromators and Hamamatsu photon multipliers (Hamamatsu PMTs type R928 and H10330B-75 were used for UV/VIS and NIR, respectively). Higher orders of the Bragg gratings were suppressed, when necessary, using a set of filters. All optical measurements were carried out on bulk materials. The prepared samples’ surfaces were smooth. Therefore, no additional polishing was performed before transmission measurements; only samples were cleaned with ethanol/acetone if needed. Materials’ thickness was in the range of 0.7–0.9 mm. Taking it into account did not change the conclusions from the measurements. It only reduced the visibility of the peaks in the transmission studies when calculated into absorption coefficient.

XPS method was used to determine the relative concentration of Eu$^{2+}$/Eu$^{3+}$. Measurements were carried out with Escalab 250 Xi spectroscope from Thermofisher Scientific. Avantage software was used for studies analysis and deconvolution. Measuring equipment has Al$_{K\alpha }$ source with pass energy 20 eV and spot size diameter 650 $\mu$m. 50 scans were performed for each element. Charge compensation was controlled through the low-energy electron and low energy Ar$^+$ ions emission by means of a flood gun, with normalization of the X-axis (binding energy) for the peak characteristics of neutral carbon C 1s (284.8 eV)^42,43^. Measurements were carried out on the ’fresh’ cross-section of each sample—samples were broken right before performing scans.

## Data Availability

All data generated or analysed during this study are included in this published article.
